# Maximizing cohesion and separation for detecting protein functional modules in protein-protein interaction networks

**DOI:** 10.1371/journal.pone.0240628

**Published:** 2020-10-13

**Authors:** Kuo-Ching Ying, Shih-Wei Lin

**Affiliations:** 1 Department of Industrial Engineering and Management, National Taipei University of Technology, Taipei, Taiwan; 2 Department of Information Management, Chang Gung University, Taoyuan, Taiwan; 3 Linkou Chang Gung Memorial Hospital, Taoyuan, Taiwan; 4 Ming Chi University of Technology, Taipei, Taiwan; Beijing University of Posts and Telecommunications, CHINA

## Abstract

Protein Function Module (PFM) identification in Protein-Protein Interaction Networks (PPINs) is one of the most important and challenging tasks in computational biology. The quick and accurate detection of PFMs in PPINs can contribute greatly to the understanding of the functions, properties, and biological mechanisms in research on various diseases and the development of new medicines. Despite the performance of existing detection approaches being improved to some extent, there are still opportunities for further enhancements in the efficiency, accuracy, and robustness of such detection methods. Based on the uniqueness of the network-clustering problem in the context of PPINs, this study proposed a very effective and efficient model based on the Lin-Kernighan-Helsgaun algorithm for detecting PFMs in PPINs. To demonstrate the effectiveness and efficiency of the proposed model, computational experiments are performed using three different categories of species datasets. The computational results reveal that the proposed model outperforms existing detection techniques in terms of two key performance indices, i.e., the degree of polymerization inside PFMs (cohesion) and the deviation degree between PFMs (separation), while being very fast and robust. The proposed model can be used to help researchers decide whether to conduct further expensive and time-consuming biological experiments and to select target proteins from large-scale PPI data for further detailed research.

## Introduction

Research on detecting Protein Function Modules (PFMs) has become one of the most important research topics in both life sciences and computing sciences since the completion of the human genome project. A PFM is a protein complex formed by groups of functionally associated proteins through disulfide bonds or other protein interactions, while a protein is made up of a linear sequence of 20 kinds of amino acids. The activity catalysis, signal transduction, and energy transportation in biological cells are the result of interactions based on groups of proteins involved in the same cell. Protein-Protein Interactions (PPIs) can initiate or inhibit a specific function within a protein complex, have a significant impact on cell function, carry out the metabolism of the organism, and even become a major cause of many diseases [1]. A protein complex in Protein-Protein Interaction Networks (PPIN) is a biomolecule relationship network consistent in both function and structure, which means the closely connected protein areas in PPINs correspond to protein functional modules. Consequently, by detecting the compactly connected structures of PPINs, it is possible to identify PFMs in order to understand cellular organization, processes, and functions [2]. More importantly, this assists in further research on various diseases, and the development of new medicines.

Given the significance of detecting PFMs in PPINs in both theory and application, this topic has attracted significant research attention. Early researchers focused mainly on biological experimental technologies, such as the yeast two-hybrid system [3], and affinity purification followed by mass spectrometry [4]. This type of detection method usually predicts the functions of the proteins by analyzing their physical interactions, properties, and chemical characteristics. However, these methods have the disadvantages of a long inspection cycle, and is expensive and time-consuming, especially when dealing with a large-scale PPIN. In recent years, due to advancements in high-throughput experiment methods, PPI datasets have become increasingly more available. With the rapid expansion of PPI data, the properties and functional modules of many protein complexes are currently unknown. Since experimental approaches are not up to the task, the development of new fast and accurate computational approaches for detecting PFMs in PPINs is of particular importance in the post-genomic era.

To address this challenge, many PFM detecting methods have been proposed in recent years. Using methodologies such as machine learning, network analysis, graph theory, and complex network theory to identify clusters of interacting proteins can help researchers gain a deeper understanding of PFMs and their evolutionary relationships. Such computational approaches can not only make up for the shortcomings of biological experimental technologies, but can also help in understanding complex higher-level cell tissues, predicting the function of unknown proteins, studying the pathogenesis of diseases, and finding new drug targets. These new computing approaches have yielded fruitful research results and progress in PFM detection.

Despite the performance of existing detection approaches being improved to some extent, there are still opportunities for further enhancements in the efficiency, accuracy, and robustness of such detection methods. Since the Lin-Kernighan-Helsgaun (LKH) [5] algorithm is one of the most effective algorithms for solving the Travelling Salesman Problem (TSP), this study combines the LKH algorithm with biological gene ontology knowledge to develop an effective and efficient model, called the Lin-Kernighan-Helsgaun Model (LKHM) for detecting PFMs in PPINs. To the best of the authors’ knowledge, this study is the first to propose a PFM detection model based on the LKH algorithm.

The remainder of this paper is organized as follows. Section 2 conducts a detailed literature review of existing detection methods, describing the progress and challenges of current research. Section 3 describes in detail the procedures for implementing LKHM. Section 4 discusses the experimental results on three commonly used benchmark sets of species and conducts performance comparisons of the proposed approach with those of existing techniques by critical measurements. Finally, Section 5 provides concluding remarks, with suggestions for future research on detecting PFMs in PPINs.

## Literature review

In the last decade, a vast amount of large-scale PPI data has been acquired using advanced approaches. These PPI data provide a good opportunity to understand PFMs for revealing unknown diseases and discovering novel therapeutic interventions. Since biological high-throughput experimental technologies remain limited by their high cost and time-consuming application, developing effective and efficient computational approaches for detecting PFMs in PPINs has become an essential and challenging problem in computational biology [6]. A PPIN is a hierarchical and modular network, which can be typically represented by a connection graph, where the individual proteins and the interactions between proteins correspond to nodes and edges in the network, respectively [7]. The weights on the edges can usually be designated as the properties of the PPIN, such as functional features. A functional module, also called a cluster, in a PPIN appears as a group of densely interconnected nodes. Therefore, identifying clusters/subgraphs from PPINs by topology-based methods makes it possible to identify families of proteins with similar functions to help researchers predict and understand protein functions and their evolutionary relationships with each other.

Despite the computational complexity of the problem, a wide spectrum of topology-based methods has been proposed for detecting PFMs in PPINs. Recent studies have shown that using topology-based methods to cluster PPINs is an effective approach for identifying PFMs. From the literature [8], representative topology-based methods can be classified into six broad categories: hierarchical-based methods, density-based methods, partition-based methods, flow-based methods, spectral-based methods, and intelligent algorithm-based methods. The progress and challenges of the six categories of topology-based methods are discussed below.

Because of the hierarchical nature of biological networks, hierarchical-based methods are applicable to the detection of PFMs by node-based and edge-based hierarchical clustering methods. These methods iteratively merge nodes or recursively divide a graph into subgraphs to accomplish the clustering of the protein nodes. For example, Aldecoa and Marín [9] used the Jerarca suite to effectively convert networks of interacting units into a tree diagram and then predicted network modules by iterative hierarchical clustering. This approach presented alternative strategies for performing iterative hierarchical clustering, which can implement an automatic evaluation of the hierarchical trees to obtain optimal partitions. Ahn et al. [10] indicated that groups of related nodes in many PPINs often have pervasive overlap. Therefore, they proposed an edge-based hierarchical clustering method to predict overlapping PFMs. Min et al. [11] presented an ensemble hierarchical clustering framework to detect PFMs. By integrating the clusters and co-complex affinity scores from different data sources, this approach can improve the detection performance of the traditional ensemble clustering method. The hierarchical-based method is a powerful tool for analyzing complex PPINs, but the computational time required to find the minimum number of cuts usually places a significant limit on its use. Besides, it is often not easy to detect overlapping protein modules when using a node-based hierarchical clustering method.

Based on the topological property that proteins within the same protein complexes have relatively high interactions than other proteins, density-based methods can be designed to identify PFMs in PPINs by searching densely connected subgraphs. Researchers have developed a variety of density-based methods to detect PFMs in PPINs. For example, Wang and Qian [12] proposed a two-step algorithm, called Finding Low-Conductance sets with Dense interactions (FLCD), for detecting PFMs in PPINs which are densely connected inside, and well separated from other networks. This approach can effectively reduce the number of edges and the search space for identifying subgraphs. Experiments on four large-scale PPINs demonstrated that FLCD outperformed the state-of-the-art algorithms. Other well-known density-based methods include Molecular Complex Detection (MCODE) [13], Protein Complex Prediction (PCP) [14], and Module Identification in Networks (MINE) [15]. The main advantage of density-based methods is their ability to find high-density protein clusters without considering low-density clusters, which is very efficient for detecting densely connected groups of proteins within a PPIN. Nevertheless, how to cluster either the sparsely connected or the relatively isolated protein nodes are the key issues of this type of method.

PPINs have a module organization structure, which is composed of PPIN topology or functionally independent subgraphs. Partition-based methods try to divide a PPIN into different modules/clusters under the assumption that the number of clusters in the network is determined in advance. The objective is to partition the network into different clusters with the largest similarity between proteins in the same cluster, and the smallest similarity between proteins in different modules. For example, Dunn et al. [16] proposed an automated partition-based method, called edge-betweenness algorithm, for separating PPINs into clusters/subgraphs of interconnected proteins, and retrieving protein annotations associated with these protein clusters. This method can rapidly predict PFMs of small- to medium-size PPINs and can resist the existence of false-positive interactions. Vlasblom and Wodak [17] compared the performance of two successful clustering procedures, Affinity Propagation (AP) and Markov Clustering (MCL), for detecting PFMs in PPINs. Experimental results showed that the MCL procedure is more tolerant to noise, and behaves more robustly than the AP algorithm. Pizzuti and Rombo [18] integrated Restricted Neighborhood Search Clustering (RNSC) with the genetic approach to detect PFMs in PPINs. Experimental results showed that the clusters obtained by the genetic approach are more accurate than those found by RNSC, though this method predicts more true complexes. Partition-based methods are very simple and easy to understand. However, this type of method usually requires prior knowledge of the exact number of clusters in the PPIN, and the clustering results depend on the quality of the initial partitions. Besides, how to detect overlapping PFMs in PPINs is still a problem that needs to be overcome in such methods.

Flow-based methods simulate the biological or functional flow in a PPIN, in which clustering is achieved by a series of flow “expansions” and “contractions” to identify clusters with high intra-cluster flows and weak inter-cluster flows. For example, Van Dongen [19] proposed a Markov Cluster (MCL) algorithm to simulate and calculate the probability of random walking nodes for predicting PFMs in PPINs. Hwang et al. [20] proposed a Signal Transduction Model (STM), for detecting PFMs in PPINs. The STM selects representative proteins for each cluster and iteratively refines clusters based on a combination of the signal transduced and graph topology to predict PFMs. The experimental results showed that STM could effectively detect both densely and sparsely connected, biologically relevant PFMs with fewer discards, and its performance was superior to the other six competing approaches. Given that there is often a significant overlap of proteins across PFMs, Shih and Parthasarathy [21] introduced a Soft Regularized Markov Cluster (SR-MCL) algorithm to address this limitation, which often leads to an impedance mismatch problem in MCL. The computational results showed that R-MCL outperformed state-of-the-art approaches in terms of accuracy of identifying PFMs in PPINs. Ochieng et al. [22] proposed a graph clustering method based on the MCL algorithm to identify PFMs in highly interconnected PPINs. The results of simulations using human proteins associated with type II diabetes mellitus revealed that this method was very reliable and efficient for detecting PFMs in PPINs. Current available flow-based methods usually do not emphasize intra-cluster connections and node density, thereby avoiding small-scale or only one node clustering results. However, some proteins will be discarded during the clustering process, and, because the information flow of all nodes must be considered, the time complexity of this type of method is usually very high.

Spectral-based methods mainly apply matrix theory and linear algebra theory to detect PFMs in PPINs. Over the last decade, several spectral clustering-based methods have been applied in complex networks and biological networks. Kamp and Christensen [23] investigated the relationship between a network’s spectral properties and its structural features within a case study on the PPIN of Drosophila melanogaster. This case study showed that the discrete part of the spectral density corresponds to the PPIN’s topological features, which could offer important insight into a network’s structure in a less biased and more systematic way than currently available. Qin and Gao [24] proposed a spectral-based method for detecting PFMs in PPINs which can determine the number of clusters based on the properties of a network. Experimental results on PPINs from DIP data and MIPS data showed that the number of clusters found by the proposed method could improve the clustering quality. The performance of the proposed spectral-based method is comparable to several other typical PFM detection algorithms. To address the heterogeneous or scale-free properties of PPINs, Inoue et al. [25] proposed an adjustable diffusion matrix-based spectral clustering (ADMSC) algorithm. The ADMSC algorithm analytically solves the clustering structure of PPINs as a problem of random walks in the diffusion process in networks. Computational results revealed that ADMSC can effectively partition PPINs into biologically significant clusters with almost equal sizes while being very fast, robust, and appealingly simple. The limitations of spectral-based methods lie in the fact that the initialization of clusters, the number of clusters, the adjacency matrix, and the choice of feature vectors will directly affect their solution quality. Besides, overlapping clusters cannot usually be predicted by spectral-based methods. Therefore, how to determine these data and predict overlapping clusters are still key issues to be solved.

The intelligent algorithm-based method is a global probability search algorithm, which considers each available solution as a biological entity. The search and optimization processes of intelligent algorithm-based methods utilize evolutionary processes based on biological behavior to identify clusters. For example, Sallim et al. [26] presented an Ant Colony Optimization (ACO) algorithm combined with the Traveling Salesman Problem (TSP), called ACOPIN, to predict PFMs in PPINs. The authors showed that ACOPIN is a feasible approach for detecting PFMs in PPINs. Lei et al. [27] combined the Firefly Algorithm (FA) and Synchronization-based Hierarchical Clustering (SHC) algorithm to detect PFMs in PPINs. They used SHC and FA to perform clustering, and to determine the optimal threshold of the neighborhood radius of synchronization, respectively. The testing and analysis results revealed that this method is superior to traditional algorithms in terms of precision, recall, and f-measure value. However, the running time of the algorithm was too long to process large-scale data. Since Particle Swarm Optimization and Swarm Optimization related algorithms have been effectively applied in solving different NP-hard problems, Zheng et al. [28] proposed an Enhanced Particle Swarm Optimization (EPSO) algorithm and a Simplified Swarm Optimization (SSO) algorithm to cluster proteins; and then introduced the knowledge of Gene Ontology (GO) for further identifying PFMs and improving detection accuracy. The results of experiments conducted on four different categories of species datasets showed that SSO is superior to EPSO in terms of prediction accuracy and solution efficiency. It can be seen from the above research that intelligent algorithm-based methods have been effectively applied to PFM detection, and the solution performance is very good. However, it remains to determine how to improve the detection efficiency and appropriately introduce biological information to improve detection accuracy.

In conclusion, many studies have shown that clustering PPINs by different topology-based methods were effective approaches for identifying PFMs. The reader is referred to the comprehensive survey papers by Wang et al. [29], Ji et al. [1], Sourav et al. [30], and Yang [8] for in-depth comparisons of various PFM detection methods. Using these topology-based methods to predict PFMs in PPINs can avoid the weaknesses of using experimental-based methods. These studies have laid a very important foundation for the theoretical development and practical application of PFM detection problems. However, as mentioned above, different topology-based methods may have their advantages and limitations. Especially, time complexity, accuracy, and robustness will still be important practical requirements that need to be improved by topology-based methods. Along with the growth of the practical needs of bioinformatics in the post-genomic era, no doubt developing robust topology-based methods will be a necessity and continuously attract attention from the bioinformatics communities for future research of PFM detection. Since the LKH algorithm has the advantages of low time complexity, high accuracy, and high robustness [5], this study combines it with biological GO knowledge to propose LKHM for detecting PFMs in PPINs.

## Proposed PFM detection model

**Fig 1:**
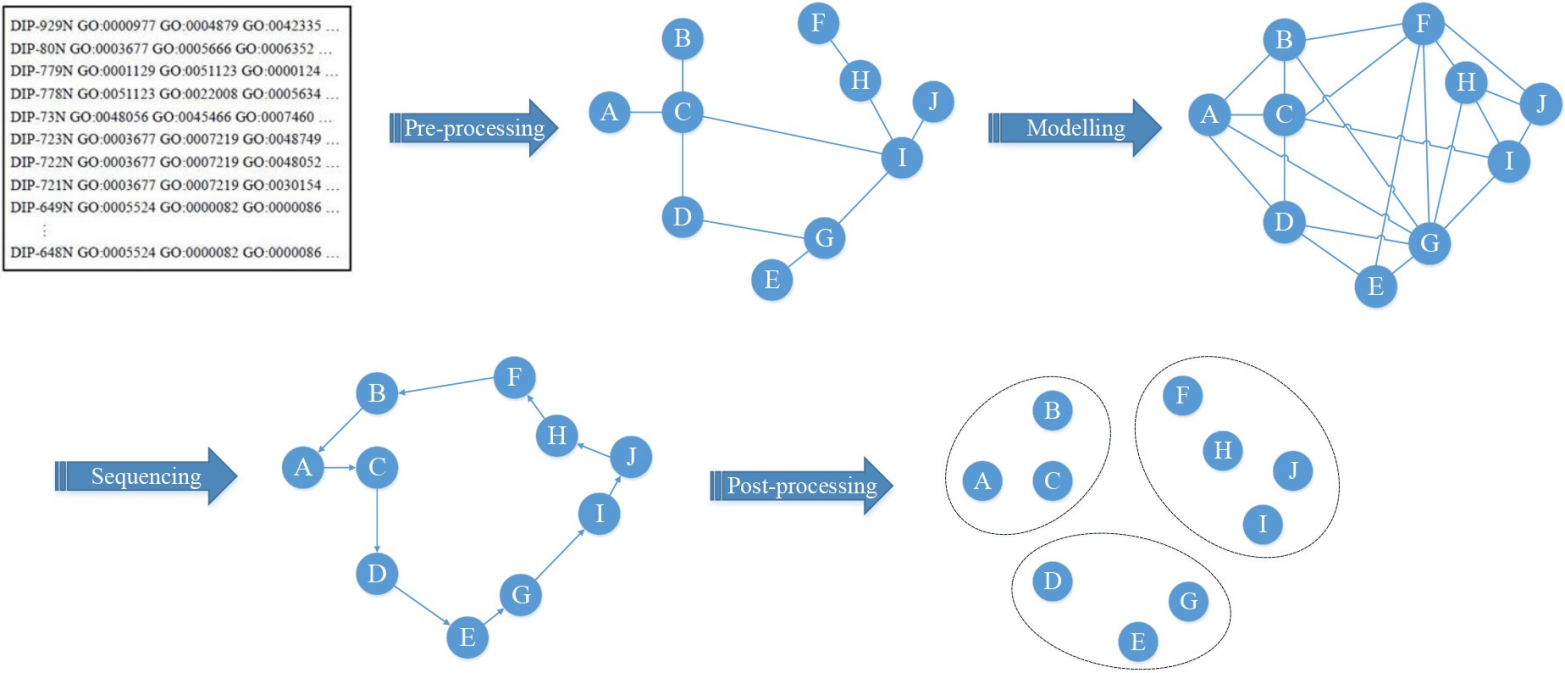
Main stages of LKHM.

To improve the time complexity, accuracy, and robustness of the PFM detection algorithm, this study proposes a new model, called the Lin-Kernighan-Helsgaun Model (LKHM), which combines the LKH algorithm with biological gene ontology knowledge for detecting PFMs in PPINs. The sketch map of LKHM is shown in Fig 1. As depicted in Fig 1, the complete PFM detection process of LKHM is composed of four main stages: data pre-processing, the PPIN connection graph modelling, the shortest path sequencing, and the clustering results post-processing. In Stage 1, the datasets acquired from Database of Interaction Proteins (DIP) [31] and Gene Ontology (GO) [32,33] are pre-processed to transform the format into a protein distance matrix. Then, in Stage 2, the PPIN connection graph is modelled using distances between proteins. In Stage 3, the shortest path of the PPIN connection graph is found using the revised LKH algorithm. Finally, the preliminary clustering results are post-processed using biological gene ontology knowledge to form the PFMs with biological meaning in Stage 4. The following subsections describe in detail the four stages of LKHM.

### 1. Data pre-processing

To tackle the problem of the incompleteness and noise of the PPIN data, the datasets acquired from existing databases must be pre-processed by three phases: noise filter, feature selection, and feature extraction, and reformat. In the noise filter phase, the crawled data with noises such as the existence of errors, blank, redundant or abnormal data must be filtered before further processing. After the noise filter, feature data are selected through the manual inspection of protein xml data in the feature selection phase. Finally, in the feature extraction and reformat phase, protein id and interactor id data are extracted and reformatted before being stored in a structured dataset.

The performance of LKHM is compared with that of EPSO and SSO algorithms [28], which are the two state-of-the-art algorithms of intelligent algorithm-based methods. To the best of the authors’ knowledge, EPSO and SSO algorithms are the current best-performing intelligent algorithm-based methods in the literature. To compare the performance of the proposed LKHM on the same basis, the experiment in this study is conducted on three species datasets, i.e., fruitfly, mouse, and human, as used by EPSO and SSO algorithms. The three species datasets are acquired from DIP and GO databases and pre-processed by Zheng et al. [28]. Table 1 lists the statistics of the three pre-processed datasets. To avoid changes in the test data version and ensure the reproducibility of the experiment, all the pre-processed datasets, protein distance matrixes of PPINs, and identified PFMs related to this study are available from http://swlin.cgu.edu.tw/data/PPIN-LKH/.

**Table 1:** Statistics of the three pre-processed datasets.

	Before Pre-processing			After Pre-processing
Species	Interaction	Interactor	GO Annotation	Interactor & GO Annotation
Human	8,412	4,823	20,201	3,394
Mouse	2,498	2,259	1,480	1,447
Fruitfly	680	607	3,299	269

### 2. PPIN connection graph modelling

Based on the topological structure of PPINs, clustering proteins in PPINs can be transformed to search the optimal tour in a connection graph, where nodes correspond to individual proteins, edges connecting two nodes correspond to interactions between proteins, and the distance between two nodes corresponds to the difference between two proteins. Nodes joined by an edge are said to be adjacent. To calculate the distance matrix of a PPIN, the adjacency matrix of interacting proteins is used. If there is an interaction between two proteins, the corresponding value in the adjacency matrix is labeled 1; otherwise, it is labeled 0. Based on the topology structure of the PPINs, the distance between nodes *i* and *j*,*d*_*ij*_ (∀*i*,*j* = 1,2,…,*n*; *i*≠*j*), can be transformed from the adjacent matrix using the following equation of Czekanowski-Dice distance (CD-Distance) [34]:

$$d_{ij}=\frac{\#(Int(i)\cup Int(j))-\#(Int(i)\cap Int(j))}{\#(Int(i)\cup Int(j))+\#(Int(i)\cap Int(j))}$$ (1)

where # represents 'number of ', and *Int*(*i*) and *Int*(*j*) denote the adjacency lists of proteins *i* and *j*, respectively.

The CD-distance is a neighborhood-based similarity measure for clustering PPINs, which has been previously employed by EPSO and SSO algorithms [28]. This distance measure has already proved its effectiveness in delineating PFMs derived from the analysis of PPINs [34]. To compare the performance of the proposed LKHM with these two state-of-the-art algorithms on the same basis, we also implement the CD-distance in this study.

**Fig 2:**
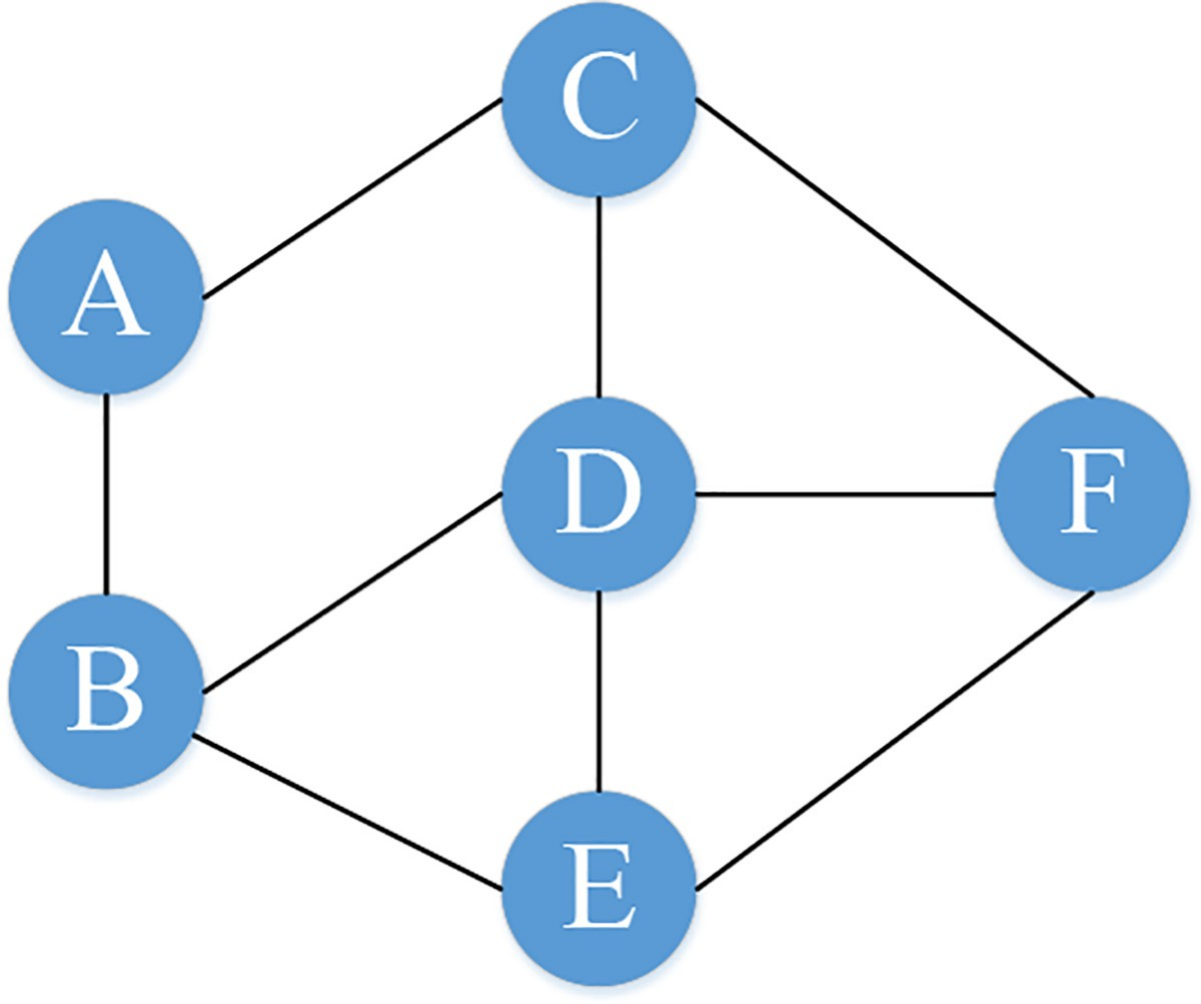
An example diagram of PPIN.

Taking the PPIN in Fig 2 as an example, the corresponding adjacent matrix and distance matrix are shown in Tables 2 and 3. If protein *i* and protein *j* both interact with the same other proteins then the distance between them will be 0. For instance, if protein *i* interacts with proteins X, Y, and Z. Meanwhile, protein *j* also interacts with proteins X, Y, and Z. Then, #(*Int*(*i*)∪*Int*(*j*)) = #(*Int*(*i*)∩*Int*(*j*)) and the distance between protein *i* and protein *j* will be 0. When proteins *i* and *j* interact with completely different sets of proteins. Then, #(*Int*(*i*)∩*Int*(*j*)) = 0 and the distance between protein *i* and protein *j* will arrive at the largest value 1. In other cases, 0<*d*_*ij*_<1. Proteins that have short distances between them are likely to jointly perform a certain function. The closer the proteins are on the shortest path, the more potential they locate in the same PFM. Therefore, the shortest path through all proteins can be interpreted as a functional clustering for all proteins in a PPIN, and then the revised LKH algorithm described in the following subsection can be employed to detect PFMs in a PPIN.

**Table 2:** Adjacency matrix.

Protein	A	B	C	D	E	F
A	0	1	1	0	0	0
B	1	0	0	1	1	0
C	1	0	0	1	0	1
D	0	1	1	0	1	1
E	0	1	0	1	0	1
F	0	0	1	1	1	0

**Table 3:** Distance matrix.

Protein	A	B	C	D	E	F
A	-	1.0000	1.0000	0.3333	0.6000	0.6000
B	1.0000	-	0.3333	0.7143	0.6667	0.3333
C	1.0000	0.3333	-	0.7143	0.3333	0.6667
D	0.3333	0.7143	0.7143	-	0.4286	0.4286
E	0.6000	0.6667	0.3333	0.4286	-	0.6667
F	0.6000	0.3333	0.6667	0.4286	0.6667	-

### 3. The shortest path sequencing

**Fig 3:**
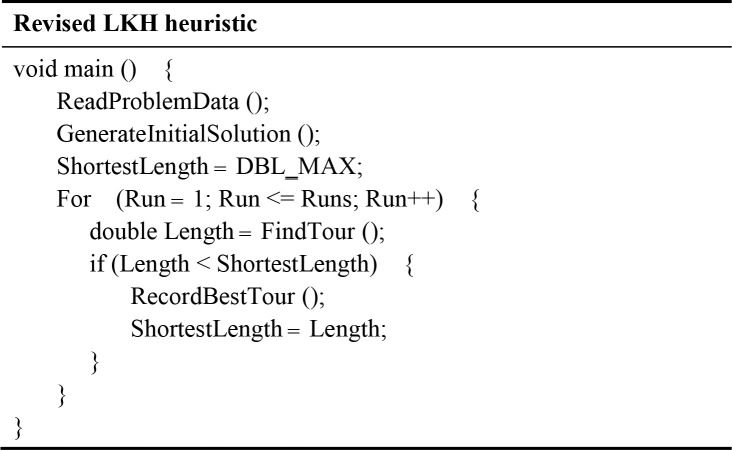
A sketch of the revised LKH algorithm.

After modelling the PPIN as a connection graph, the revised LKH algorithm is implemented to search the shortest path of the graph. As shown in Fig 3, the revised LKH is composed of two phases. In the first phase, the nearest neighbour (NN) rule [35] is used to generate an initial tour for the PPIN. Subsequently, the LKH algorithm [5] is imported to improve upon the obtained initial tour and searches the shortest path of the PPIN connection graph in the second phase. The two phases of the revised LKH algorithm are described in detail below.

**Phase I**: After reading the specification of the problem to be solved, use the nearest neighbour (NN) rule to generate an initial tour.

*Step 1*.*1*: Start from a dummy node as the current vertex.

*Step 1*.*2*: Find the shortest edge between the current vertex with an unvisited vertex *V*, and add vertex *V* to the current tour.

*Step 1*.*3*: Update the current vertex to *V*, and mark *V* as a visited node.

*Step 1*.*4*: If all of the vertices in the PPIN connection graph have been visited, then terminate and set the current tour as an initial tour; otherwise, go back to *Step 1*.*2*.

**Phase II**: Apply the LKH algorithm to search the shortest path of the PPIN connection graph.

*Step 2*.*1*: Implement six partitioning schemes (i.e., tour segment partitioning, Karp partitioning, Delaunay partitioning, K-means partitioning, Sierpinski partitioning, and Rohe partitioning) to partition the problem into smaller sub-problems.

*Step 2*.*2*: Set the initial tour as the incumbent tour, and set the length of the incumbent tour to a large floating-point number (denoted as DBL_MAX). Repeatedly perform the following procedures for a specified number of runs to improve the incumbent tour.

        *Step 2*.*2*.*1*: Repeatedly apply five revised Lin and Kernighan’s criteria (i.e., the sequential exchange criterion, the feasibility criterion, the positive gain criterion, the disjunctive criterion, and the candidate set criterion) to execute the general K-opt moves on the sub-problems to reduce their lengths until no exchange can shorten the sub-tours, where K is an integer chosen from the interval [2,*n*].

        *Step 2*.*2*.*2*: Use the tour merging procedure to produce the best possible tour from the given sub-tours.

Owing to space limitations, this paper does not provide details of the six partitioning schemes, the five revised Lin and Kernighan’s criteria, the general K-opt moves, and the tour merging procedure of the LKH algorithm. The reader is referred to the paper by Helsgaun [5] for a detailed discussion of the LKH algorithm.

### 4. Clustering results post-processing

To improve the accuracy of clustering results, the preliminary clustering results are post-processed using biological gene ontology knowledge. In this step, the following post-processing procedures of function information-based PFM optimization and topology-based PFM optimization are executed, respectively, to form the final PFMs with biological meaning.

### 4.1 Function information-based PFM optimization

The purpose of the function information-based PFM optimization is to iteratively merge modules that are functionally close by evaluating the similarity of two PFMs of the preliminary clustering results. The functional similarity of any two PFMs *M*_*A*_ and *M*_*B*_, *S*(*M*_*A*_,*M*_*B*_), is measured by the following equation [28]:

$$S(M_{A},M_{B})=\frac{\sum_{i\in M_{A},j\in M_{B}}s(i,j)}{\min\{|M_{A}|,|M_{B}|\}}$$ (2)

where |*M*_*A*_| and |*M*_*B*_| represent the number of proteins in PFMs *M*_*A*_ and *M*_*B*_, respectively, and *s*(*i*,*j*) is the similarity parameter of two proteins *i* and *j* that belong to *M*_*A*_ and *M*_*B*_, respectively, which can be calculated by the equation as follows:

$$s(i,j)=\left\{ \begin{array}{l} 1,\;\mathrm{if}\;i=j\\ \frac{\left|g^{i}\cap g^{j}\right|}{\left|g^{i}\cup g^{j}\right|},\;\mathrm{if}\;i\neq j \end{array}\right.$$ (3)

where *g*^*i*^ and *g*^*j*^ denote the comment values of protein *i* and *j* in the GO, respectively.

In this step, if the functional similarity of any two PFMs *M*_*A*_ and *M*_*B*_, *S*(*M*_*A*_,*M*_*B*_), is greater than a predefined threshold, *θ*, then *M*_*A*_ and *M*_*B*_ will be merged into one PFM. The merge process continues until all cluster pairs satisfy the predefined threshold.

### 4.2 Topology-based PFM optimization

To further improve the accuracy of clustering results, PFMs with lower densities are filtered and discarded using a lower boundary of the module density. The module density can be measured by the following equation:

$$D_{s}=\frac{e}{N\times (N-1)/2}$$ (4)

Where *N* represents the number of current PFMs and *e* denotes the number of interactions in the module.

## Experiment results

This section describes the experiments conducted in this study to evaluate the performance of the proposed LKHM in detecting PFMs from PPINs. The proposed LKHM was coded in Java and C++ programming languages. The revised LKH algorithm was implemented by C++ in Visual Studio 2010, while the other part of LKHM was implemented by java, and was executed using Eclipse 8.0. All experiments were conducted on a personal computer with an Intel® Xeon® E5-1620 v2 processor running at 3.70 GHz, with 64 GB of RAM. The following subsections describe the performance evaluation indices and compare the computational results obtained by LKHM with those obtained using the other two state-of-the-art algorithms on three species datasets.

### 1. Performance evaluation indices

To compare the performance of LKHM with EPSO and SSO algorithms on the same basis, two performance evaluation indices, cohesion (*C*_*o*_) and separation (*S*_*e*_), which were used by Zheng et al. [28] to evaluate EPSO and SSO algorithms, are applied in this study. The values of *C*_*o*_ and *S*_*e*_ are calculated by the following equations:

$$C_{o}=\left\{ \begin{array}{l} S+\frac{1}{D},\;D\neq 0\\ S,\;D=0 \end{array}\right.$$ (5)

$$S_{e}=\left\{ \begin{array}{l} D+\frac{1}{S},\;S\neq 0\\ D,\;S=0 \end{array}\right.$$ (6)

where *S* and *D* denote the values of nodes in the functional similarity matrix and distance matrix, respectively.

Cohesion is a measure that denotes the degree of polymerization inside PFMs. The higher the value of *C*_*o*_, the higher the similarity degree of proteins in the same PFMs. Separation refers to the deviation degree between PFMs. The higher the value of *S*_*e*_, the higher the dissimilarity between different PFMs.

### 2. Analytical results and discussion

**Fig 4:**
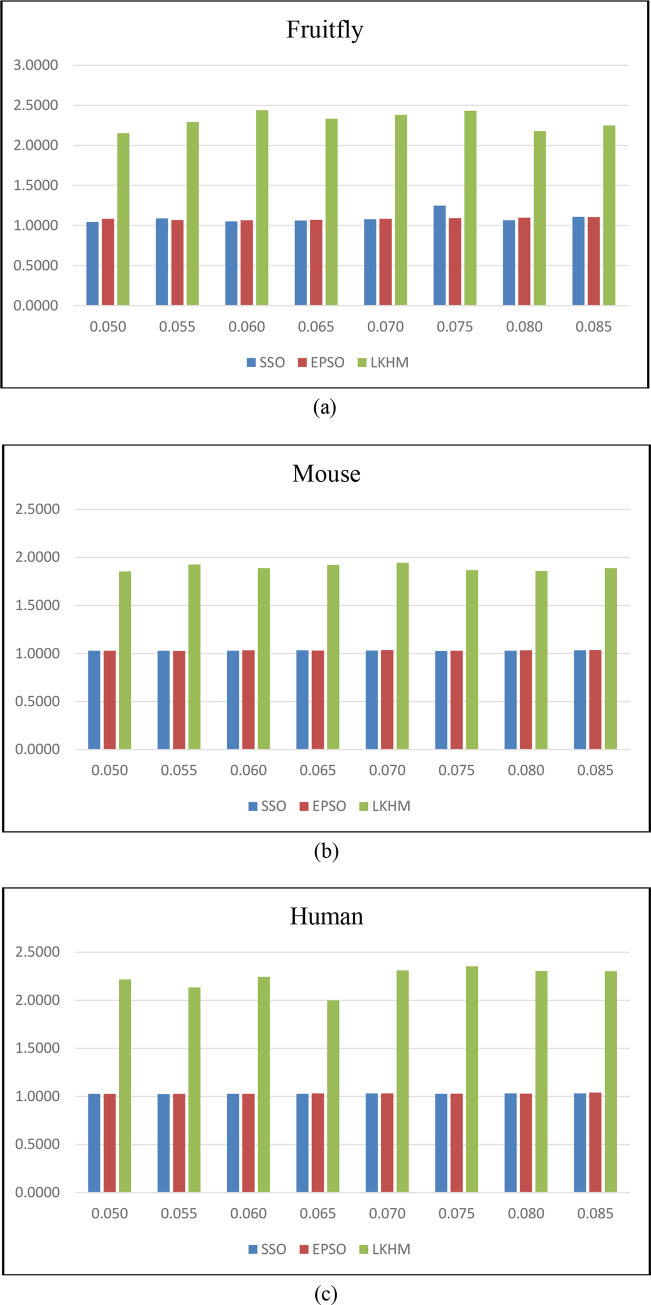
Degrees of polymerization inside PFMs.

For the three species datasets, the statistics results of *C*_*o*_ and *S*_*e*_ for the SSO, EPSO, and LKHM under eight different threshold values are summarized in Tables 4 and 5. As revealed in Table 4, when applied to the fruitfly, mouse, and human datasets, the total average values of *C*_*o*_ obtained by SSO, EPSO, and LKHM were (1.0940, 1.0302, 1.0281), (1.0835, 1.0319, 1.0304) and (2.3073, 1.8937, 2.2324), respectively. On the other hand, as demonstrated in Table 5, the total average values of *S*_*e*_ obtained by SSO, EPSO, and LKHM for the fruitfly, mouse, and human datasets were (19.9421, 23.4273, 22.9155), (20.3769, 23.8979, 22.8809), and (21.0179, 22.9875, 22.0317), respectively. The degree of polymerization inside PFMs obtained by LKHM is significantly higher than that obtained by the SSO and EPSO approaches, whereas the deviation degree between PFMs obtained by LKHM remained nearly at the same level as that obtained by SSO and EPSO approaches. That is, the similarity degree of proteins in the same PFMs generated by LKHM is significantly higher than that generated by the SSO and EPSO approaches, especially for the fruitfly dataset; while the dissimilarity degree of proteins between different PFMs of the three compared approaches are approximately the same. These analytical results can also be seen in Fig 4. This improvement is significant, demonstrating that PFMs can be identified from PPINs more accurately using LKHM.

**Table 4:** Degrees of polymerization inside PFMs (*C*_*o*_) for compared approaches.

	SSO			EPSO			LKHM		
Threshold	Fruitfly	Mouse	Human	Fruitfly	Mouse	Human	Fruitfly	Mouse	Human
0.050	1.0448	1.0280	1.0270	1.0842	1.0288	1.0272	2.1514	1.8548	2.2176
0.055	1.0906	1.0298	1.0242	1.0688	1.0278	1.0276	2.2928	1.9256	2.1338
0.060	1.0516	1.0296	1.0278	1.0666	1.0334	1.0274	2.4402	1.8886	2.2436
0.065	1.0634	1.0326	1.0276	1.0710	1.0310	1.0310	2.3326	1.9212	2.0004
0.070	1.0778	1.0306	1.0308	1.0852	1.0356	1.0312	2.3818	1.9446	2.3118
0.075	1.2486	1.0274	1.0276	1.0930	1.0296	1.0288	2.4328	1.8684	2.3538
0.080	1.0658	1.0300	1.0302	1.0980	1.0334	1.0296	2.1786	1.8596	2.3036
0.085	1.1094	1.0342	1.0302	1.1050	1.0362	1.0408	2.2484	1.8868	2.3032
Total Ave.	1.0940	1.0302	1.0281	1.0835	1.0319	1.0304	2.3073	1.8937	2.2324

**Table 5:** Deviation degrees between PFMs (*S*_*e*_) for compared approaches.

	SSO			EPSO			LKHM		
Threshold	Fruitfly	Mouse	Human	Fruitfly	Mouse	Human	Fruitfly	Mouse	Human
0.050	19.9450	23.8480	22.8452	21.1064	23.9212	22.7966	18.9636	22.8962	21.5276
0.055	20.3240	23.6394	23.0308	20.5620	24.0596	22.8374	19.1828	22.6266	21.9868
0.060	19.6422	23.5466	23.1240	19.6758	24.0378	23.0052	20.5374	22.8688	21.7224
0.065	20.1214	23.6064	22.8264	20.0156	24.0522	22.5876	21.8196	23.4734	21.9264
0.070	19.7058	23.2210	22.9818	20.5950	23.9542	22.9120	21.9656	22.9996	22.4988
0.075	19.5328	23.5762	22.8524	20.3126	23.6772	22.7920	21.9108	22.9906	22.2826
0.080	20.4202	23.2104	22.9208	20.0296	23.7938	23.0494	21.9372	23.1178	22.1316
0.085	19.8458	22.7710	22.7432	20.7184	23.6878	23.0670	21.8264	22.9274	22.1776
Total Ave.	19.9421	23.4273	22.9155	20.3769	23.8979	22.8809	21.0179	22.9875	22.0317

Table 6 shows the average computational times (CPU times in seconds) spent by the three compared approaches when applied to each species dataset. As seen in Table 6, when applied to the fruitfly, mouse, and human datasets, the total average computational times required by LKHM were 0.7 s, 71.5 s, and 421.2 s, respectively; whereas the corresponding values required by SSO were 4.0 s, 78.2 s, and 460.5 s, respectively, and the corresponding values required by EPSO were 4.9 s, 161.8 s and 699.0 s, respectively. Since computational times may vary with different hardware, software, and programming skills, it is not very fair to directly compare the efficiency of the SSO, EPSO, and LKHM approaches based on these experimental results. However, the average computational times listed in Table 6 show that LKHM can cluster PFMs better than can SSO and EPSO within a very short and reasonable computation time. Notably, the computational time of LKHM is not affected by different threshold values. These results demonstrate that LKHM outperforms the two state-of-the-art approaches in detecting PFMs in PPINs in terms of cohesion, separation, and robustness.

**Table 6:** Average computational times for compared approaches.

	SSO			EPSO			LKHM		
Threshold	Fruitfly	Mouse	Human	Fruitfly	Mouse	Human	Fruitfly	Mouse	Human
0.050	4.4*	71.2	731.6	5.4	148.6	710.2	0.7	71.5	421.2
0.055	4.0	69.4	501.4	4.0	167.4	650.0	0.7	71.5	421.2
0.060	4.0	75.6	451.8	5.0	163.2	637.8	0.7	71.5	421.2
0.065	4.0	73.6	407.6	5.0	168.0	683.8	0.7	71.5	421.2
0.070	4.0	107.4	418.8	5.0	146.0	629.6	0.7	71.5	421.2
0.075	4.0	78.0	375.6	5.0	168.0	758.2	0.7	71.5	421.2
0.080	4.0	74.6	395.2	5.0	166.4	751.2	0.7	71.5	421.2
0.085	4.0	76.4	402.0	5.0	167.2	771.2	0.7	71.5	421.2
Total Ave.	4.0	78.2	460.5	4.9	161.8	699.0	0.7	71.5	421.2

* CPU time in second.

## Conclusions and recommendations for future studies

PFM identification in PPINs is one of the most important and challenging tasks in computational biology. The quick and accurate detection of PFMs in PPINs can contribute greatly to the understanding of the functions, properties, and biological mechanisms in research on various diseases and the development of new medicines. Based on the uniqueness of the network-clustering problem in the context of PPINs, this study proposed a very effective and efficient model based on the LKH algorithm for detecting PFMs in PPINs. The simulation results show that the proposed technique has superior performance in terms of cohesion and separation when compared to other existing methods. The proposed technique is a very reliable method for detecting PFMs in PPINs, which can effectively reduce the computational time and cost, and improve the accuracy of PFM detection. It can be used to help researchers decide whether to conduct further expensive and time-consuming biological experiments and to select target proteins from large-scale PPI data for further detailed research. The authors hope that this study will contribute to the theory of computational biology, and to applications in uncovering the pathogenesis of diseases and developing new drug targets.

Given the significance of the PFM detection problem in both theory and application, there is much room for further research in this field. Some important directions and interesting topics for further studies are suggested as follows. First, it is necessary to develop other effective, efficient, and robust computational methods, such as matheuristics, for detecting PFMs in PPINs. Second, the development of PFM detection approaches with high tolerance to data noises would be an interesting target of practical research. Third, the development of distributed and parallel algorithms to detect PFMs in large-scale PPINs within an acceptable computational time warrant further exploration. Fourth, new methods for combining PFM detection methods with functional annotation and biological evolution messages would support a rich body of future studies. Finally, the detection of PFMs in overlapping modules and dynamic PPINs are still in their initial stages and are worth further research.
